# Sexual and reproductive health needs assessment & interventions in a female psychiatric intensive care unit

**DOI:** 10.1192/bjo.2021.175

**Published:** 2021-06-18

**Authors:** Elizabeth Rose, Elana Covshoff, Lucy Blake, Adenike Bolade, Robert Rathouse, Aleshia Wilson, Arthur Cotterell, Rudiger Pittrof, Faisil Sethi

**Affiliations:** 1 South London and Maudsley NHS Foundation Trust; 2 Guy's and St Thomas NHS Foundation Trust; 3 Dorset Healthcare University NHS Foundation Trust

## Abstract

**Aims:**

To assess the sexual and reproductive health (SRH) needs of women admitted to a psychiatric intensive care unit (PICU), and acceptability of delivering specialist SRH assessments/interventions in this setting. Secondary aims were to explore the barriers to access and the feasibility of providing SRH assessments and interventions in the PICU.

**Method:**

A retrospective analysis of fifteen months’ activity data found that only 25 SRH referrals had been made across 205 PICU admissions. This low referral rate of 12% likely reflected pathway barriers and was unlikely to represent the actual clinical need in female PICU patients. A bi-monthly SRH in-reach clinic and a nurse led SRH referral pathway were implemented on the PICU over a seven-month period. Within a quality improvement framework, a staff training needs assessment was performed, training delivered, a protocol developed, staff attitudes explored, and patient and carer engagement sought.

**Result:**

A quality improvement approach streamlined SRH assessments on the PICU and resulted in 42% of women being assessed and a 3.5-fold increase in uptake. At least 30% of the women in the PICU had unmet SRH needs identified and proceeded to a specialist appointment. This amounts to a minimum 2.5-fold increase in SRH unmet need detection.

The most common SRH needs were complex gynaecological issues (such as period problems, pelvic pain, vaginal discharge), STI advice/testing and contraception advice/options. 21% of women initiated SRH interventions, and 14% completed all the interventions required for their needs. The most common interventions were in the areas of contraception advice/family planning and STI advice/testing.

Staff confidence on assessing SRH topics was identified as a barrier to access with a positive shift noted after bespoke SRH training was implemented and a protocol introduced: on a scale of 0-10 (with 10 being high), 81.3% of staff rated their confidence 8 or above in relation to discussing contraception/sexually transmitted infections (pre-training: 25.0%), and 93.8% in relation to discussing risky behaviours (pre-training: 18.8%). All 11 patient and carer participants felt it was important to have a forum to talk about SRH and 8 (72.7%) agreed it was important in the PICU.

**Conclusion:**

Results identify that SRH needs for PICU admissions are greater than previously realised. Staff highlighted the acceptability and importance of SRH care, if interventions are appropriately timed and the patient's individual risk profile considered. Providing a nurse led referral pathway for an SRH in-reach clinic is acceptable, feasible and beneficial for PICU patients.

